# Repurposing of End-of-Life Dialysate Production Polymeric Membrane for Achieving Sustainable Hemodialysis Process Water Management

**DOI:** 10.3390/polym17212922

**Published:** 2025-10-31

**Authors:** Nuhu Dalhat Mu’azu, Aesha H. AlAmri, Ishraq H. Alhamed, Mukarram Zubair, Mohammad Saood Manzar, Muhammad Nawaz

**Affiliations:** 1 Department of Environmental Engineering, College of Engineering, Imam Abdulrahman Bin Faisal University, Dammam 31441, Saudi Arabia; mzzubair@iau.edu.sa (M.Z.); msmanzar@iau.edu.sa (M.S.M.); 2 Department of Chemistry, College of Science, Imam Abdulrahman Bin Faisal University, P.O. Box 1982, Dammam 31441, Saudi Arabia; ahalamri@iau.edu.sa (A.H.A.); 2230700017@iau.edu.sa (I.H.A.); 3 Department of Nano-Medicine Research, Institute for Research and Medical Consultations (IRMC), Imam Abdulrahman Bin Faisal University, Dammam 31441, Saudi Arabia; mnnmuhammad@iau.edu.sa

**Keywords:** RO desalination membranes, chemical rehabilitation, response surface methodology, closed-looped sustainable wastewater treatment, fouling removal and mitigation, dialysis center effluent, health care facilities waste management, environmental sustainability, spent dialysate recovery, wastewater reclamation, resource recovery

## Abstract

Polymeric reverse osmosis (RO) membranes are critical for producing ultrapure water for hemodialysis process, but once they reach their end-of-life (EoL) stage, mainly due to fouling, they are usually discarded—adding to the growing challenges of medical waste management. This study explores a sustainable alternative by rehabilitating EoL thin-film composite (TFC) membrane and its reuse in recovery of spent dialysate. Using different cleaning agents that included citric acid (CA), EDTA, sodium lauryl sulfate (SLS), and sodium dodecyl sulfate (SDS), the mixture of CA and SLS (1:1) exhibited the most effective combination for balanced flux recovery, salt rejection, and creatinine clearance at lower TMP, achieving 90% conductivity reduction, 46.89 L/m^2^/h water flux, and 1.24 L/m^2^/h/bar permeance. FTIR, SEM, and EDX results confirmed the removal of both organic and inorganic foulants, while further process optimization revealed the critical role of cleaning temperature, SLS ratio and pressure on water permeability and improving creatinine removal. Under the optimal operational conditions, 99.89% creatinine removal, while restoring up to 80% hydraulic performance, yielding water flux and permeance of 59.36 L/m^2^/h and 1.79 L/m^2^/h/bar, respectively. These findings suggest that reduced dialysate production costs and minimize environmental impact can be significantly, achieved by extending the useful life of dialysate membranes, thereby opening a pathway toward implementing closed-loop water management and circular economy practices at dialysis centers.

## 1. Introduction

Hemodialysis is a common and important treatment for people who suffer from kidney failure [1]. Over the decades, hemodialysis has improved with the improvements in membrane technology which plays a central role in producing ultrapure water and is employed in the water treatment system to prevent risk and adverse reactions during the hemodialysis process [2,3,4] by rejecting all impurities while maintaining the chemical and microbial safety requirements for the dialysis procedure. Consequently, polymeric RO membranes are essentially used in water treatment for hemodialysis processes and many other medical applications [2,5,6] to produce ultrapure water to meet international health standards and regulatory compliance. These membranes are made of polyamide thin-film composites, which enable high permeability to be maintained and the removal of a variety of impurities including salts and organic pollutants through advanced RO technology [6]. Despite the membrane’s effectiveness, it has a limited lifespan. One of the most significant problems associated with RO membranes, particularly in the context of ultrapure water production for HD process is fouling [6,7]. Fouling of membrane happens when microorganisms, organic and inorganic matter, and particles accumulate on the membrane surface, causing reduction in water flux and permeate quality and increasing operating costs [8]. Also, the accumulation of particles and other undesirable molecules on the surface of the membrane lowers the osmotic pressure gradient. Membrane fouling may require higher frequency of cleaning. Meanwhile, chemical cleaning and extreme cleaning conditions to remove fouling can lead to damage of the polyamide layer of the RO membrane, which ultimately decreases its rejection performance and simultaneously deteriorates the permeate water quality [9].

**Figure 1:**
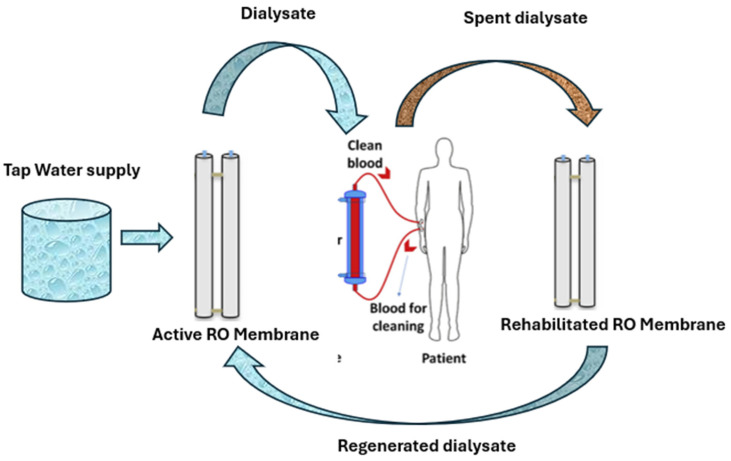
Sustainable closed-loop dialysate water management approach.

The capital and operational costs associated with RO systems are generally high [8]. This includes not only energy consumption costs but also the maintenance cost. Usually, fouled and exhausted EoL membranes are discarded in landfills or incinerated, resulting in significant waste challenges. Muazu et al. [2] in a recent review on hemodialysis polymeric membranes, advocated the adoption of a sustainable approach towards HD water management by implementing a closed-loop strategy for dialysate as illustrated in Figure 1. Globally, strategic sustainable visions -such as Saudi Arabian Vision 2030 -are aligned with UN SDGs initiatives that promote sustainable and environmentally friendly practices towards achieving circular economy [9,10,11,12,13], highlighting the need to repurpose discarded membrane for other applications such as spent dialysate wastewater treatment [2,7].

At the same time, hospitals and healthcare facilities seek cost-effective and efficient solutions for managing spent dialysate to reduce operational waste and environmental impact. Recycling and repurposing rehabilitated reverse osmosis (RO) membranes is a promising strategy, offering significant economic and environmental benefits [9]. This approach not only reduces capital investment by avoiding the purchase of new RO modules—which have high production costs—but also lowers the overall carbon footprint associated with RO membrane manufacturing. It is estimated that using chemically cleaned or converted RO membranes can yield 75% to 85% cost savings compared to purchasing a new module, thereby making the water treatment process more sustainable and affordable [9]. Currently, the market for ultrapure water production mostly uses new nanofiltration and RO modules that expensive in comparison with rehabilitated membranes. Moreover, since the regulations encourage waste reduction and the use of most resources, integrating recycled materials in industrial processes is a favorable choice. The opportunity to address environmental and economic challenges by rehabilitating discarded HD membranes cannot be further emphasized as with an optimal rehabilitation method, EoLM has the potential to be transformed into an effective alternative for treating simulated spent dialysate wastewater, which would align with global sustainability goals and decrease the dependency on landfill disposal while achieving zero liquid discharge and a circular economy [14]. This will offer a different application scalability and eco-friendly solution to water treatment needs involving hospitals, dialysis centers, and healthcare waste management sectors.

Fouling is still expected to occur despite advancements in its prevention; cleaning methods can be applied for reduction in fouling based on its type, achieving a partial recovery of the initial hydraulic permeability. Several cleaning techniques such as ultrasonic [15,16,17], chemical, and other approaches have been implemented. In this context, chemical cleaning, as the most common technique for fouled membrane cleaning, involves the use of various chemical agents to remove wide ranges of foulant from membranes after long period operations. Commonly used chemicals include acids such as hydrochloric acid, sulfuric acid, and citric acid for scaling removal [8,18,19,20,21,22,23,24], sodium hydroxide, chelating agents (such as EDTA), surfactants like sodium dodecyl sulfate (SDS) for addressing organic fouling removal while biocides such as sodium bisulfite for biofouling control [8,19,20,21,22,23,24]. Numerous studies investigated the cleaning schemes and performance of cleaned RO membranes for wastewater treatment. For instance, Khaless et al. [11] explored regenerated membranes for industrial phosphoric acid treatment from which they demonstrated that osmosis (RO) membranes could achieve a rejection of 70% for suspended solid and 61% for organic matter. In another study, seawater RO modules contaminated with silica–aluminum deposits were cleaned by two-step chemical cleaning and showed up to 96% salt rejection [18]. In a similar work, Park et al. [23] optimized the chemical cleaning process by tailoring influential factors such as chemical concentration, time, and temperature. The author reported 86% flux recovery using an optimized cleaning protocol. Meanwhile, Jung et al. [19] used mixed chemical reagent (0.5% EDTA and 0.1% SDS) for the cleaning of discarded RO membranes. The authors indicated that the lower concentration level of mixed reagents showed efficient removal of fouling from membrane surfaces and demonstrated almost >85% of salt rejection. These studies underscore the possibility of repurposed RO membranes as a cost-effective and eco-friendly solution for wastewater management applications. However, the optimization of cleaning methods and the application of green solvents for cleaning require further investigation [7]. In addition, despite the cost reduction and environmental benefits of recycling RO membranes, there remains the issue of possible degradation of the active polyamide layer of RO membranes due to exposure to chemicals in consecutive cleaning cycles, which can impact the long-term performance of recycled RO membranes. Based on the detailed literature, no work has been reported on the rehabilitation of end-of-life (REoLM) hemodialysis RO membranes and its application in the management of wastewater from the health sector.

This present study explores rehabilitation of end-of-life (REoLM) hemodialysis RO membranes, specifically tailored for the treatment of spent dialysate wastewater. Unlike previous research on repurposing RO membranes, this work uniquely targets the chemical rehabilitation of membranes previously used in dialysate production, addressing the specific challenges posed by their ultrapure water production context. The novelty lies in developing an optimal rehabilitation method via exploring different environmentally friendly, biodegradable, and common chemical agents (such as citric acid, SD, and SLS) under a low-heating environment to restore EoLM from a typical dialysate center for the remediation of creatinine-laden water—a typical spent dialysate contaminant—which is a niche application not found in the literature. Additionally, statistical optimization techniques were employed to evaluate the multi-variable performance of the rehabilitated dialysate EoLM. This approach explores the sustainability of the process while maintaining high removal efficiency, not only aligning with global sustainability goals by reducing waste but also offering a cost-effective alternative to conventional treatment methods, potentially transforming healthcare waste management practices. The study’s findings could pave the way for scalable implementation in dialysis centers, providing an environmentally and economically viable solution tailored to the unique demands of hemodialysis wastewater reuse.

## 2. Materials and Methods

### 2.1. Dialysate Center RO Membrane Unit and EoLM Sample Preparation

The EoLM module used in the present study was supplied by a hospital dialysate center at Al-khobar. The pristine membrane module was a FilmTec™ thin-film composite (TFC) polyamide membrane with original operational characteristics given in Table S1 as per the producers specifications.

### 2.2. Chemical Reagents

The primary chemical reagents used for cleaning the dialysate EoLM included EDTA, SDS, CA, and SLS from Scharlau (Barcelona, Spain) and Sigma Adrich (Saint Louis, MO, USA), which were all procured from a local supplier. All cleaning solutions were prepared by complete dissolution of the chemical agents in deionized (DI) water without any other chemical addition or further purification.

### 2.3. EoLM Rehabilitation Using Different Chemical Cleaning Agents

**Figure 2:**
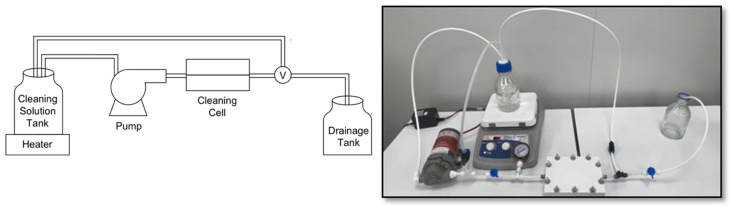
EoLM cleaning flow-cell experimental set-up.

To identify the most effective cleaning agent, a series of conductivity removal experiments were conducted to evaluate various chemical combinations for removing different types fouling found from the collected dialysate center EoLM sample. Due to its availability, been non-toxic and environmentally friendliness, CA was selected as the primary cleaning agent; then experiments were designed based on the literature findings to mix CA with other the agents. Thus, the tested cleaning agents included CA in combination with EDTA, SDS, and SLS (SDS and SLS are biodegradable, thus environmentally friendly also). The supplied EoLM module was dismembered, and the fouled polymeric TFC membrane sheet component was carefully removed and cut into sheets and rinsed with deionized water. Without any pH adjustment, each rehabilitation experiment, entails immersing the EoLM sheet in a cleaning solution in 500 mL flask and stirred on a magnetic stirrer hot plate for 30 min at a required fixed temperature. Then the cleaned sheets were rinsed with deionized water, left to air-dry and the cut into smaller pieces (4.5 × 4.7 cm^2^) to fit into the Sterlitech^@^ (Sterlitech Corporation, Washington, DC, USA) benchtop cross/tangential flow system and applied for filtration of NaCl_2_ salt solution (1037 µS/cm average conductivity) at a fixed TMP depending on the required pressure to maintained a fixed flow rate of 0.5 gal/min. Similarly, a prototype low pressure flow cell (Figure 2) was developed for the cleaning and tested which yielded comparable performances. Cleaning performance was assessed primarily through measuring the conductivity of the permeate water, followed by physicochemical characterization as detailed in the next section.

### 2.4. Physico-Chemical Characterization of EoL Membranes

Fourier Transform Infrared Spectroscopy (FTIR), Scanning Electron Microscopy (SEM), and Energy Dispersive X-ray Spectroscopy (EDX) were employed to characterize the end-of-life (EoL) membrane samples before and after chemical cleaning. FTIR spectra using an ATR-FTIR spectrometer (Perkin Elmer) was conducted in the range of 4000–500 cm^−1^ to identify changes in surface functional groups and possible chemical degradation of the membrane material. SEM analysis was performed after sputter-coating the samples with a thin layer of gold to examine surface morphology, fouling deposition, and structural integrity. Elemental composition and mapping of inorganic foulants were examined using an energy-dispersive X-ray spectrometer (EDX) equipped with SEM (TESCAN). Together, these techniques provided complementary insights into the physicochemical, morphological, and surface properties of the original and the rehabilitated EoLM.

### 2.5. Remediation of Creatinine Contaminated Water Under FCC-CCD Design

Based on the experiment assessments of the different chemical agents’ combinations on conductivity removal in Section 2.4 above, the CA and SLS mixture (1:1) was selected as the cleaning agent due to its best-balanced performance in terms of permeability and salt rejection in relation to lowered TMP, its compatibility with the membrane material, as well as its environmentally friendliness. Accordingly, the performance of the dialysate REoLM in the removal of creatinine were studied and evaluated and optimized through regression models based on response surface methodology (RSM) using a face-centered central composite design (FC-CCD). As per Table 1, the influence of operational variables A = temperature (25–65 °C), B = SLS ratio (25–75%), and C = applied TMP (350–550 psi) on performance evaluators coded as −1, 0 and +1, respectively. Four(4) key REoLM performance response variables investigated included water flux (Y1), water permeance (Y2), creatinine residual concentration (Y3) and creatinine removal efficiency (Y4) were investigated and calculated using Equations (1) to (4). The filtration tests were also undertaken using the Sterlitech^@^ benchtop cross/tangential flow filtration system.

(1) $$Y1=Water\;flux=\frac{V}{A.t}\;L/m^{2}/\mathrm{hr}$$

(2) $$Y2=Water\;permaence=\frac{V}{PA.t}\;L/m^{2}/h/\mathrm{bar}$$

(3) $$Y3=Creatinine\;residual\;concentration=C_{o}-C_{t}\;\mathrm{mg}/L$$

(4) $$Y4=Creatinine\;residual\;concentration=\left(\frac{C_{o}-C_{t}}{C_{o}}\right)\times 100\%$$

where *V* = filtered permeate volume, *A* = membrane cross flow area, *t* = filtration time, *P* = TMP, *C_o_* = initial concentration of solute, *C_t_* = final concentration of solute

### 2.6. Creatinine Concentration Measurement

Creatinine concentrations in the feed water and the permeate collected after filtration tests were determined using a high-performance liquid chromatography (HPLC) system (Ultimate 3000, Thermo Fisher Scientific, Les Ulis, France). The system was equipped with an the Thermo Scientific™ Hypersil GOLD™ Amino column (Catalog No. 25705-154630) and operated under a binary gradient mobile phase comprising 20% deionized water and 80% HPLC-grade acetonitrile. The mobile phase was filtered and degassed prior to use to ensure baseline stability. Chromatographic conditions were set as follows: flow rate 0.3 mL min^−1^, column temperature 30 °C, injection volume 10 µL, and UV detection at 220 nm. Each sample was filtered through a 0.22 µm syringe filter before analysis. Quantification was based on calibration curves prepared from standard creatinine solutions covering the working concentration range, with a linear regression of R^2^ > 0.99.

## 3. Results Discussions

### 3.1. Dialysate Production EoLM Cleaning Using Different Binary Chemical Agent

**Figure 3:**
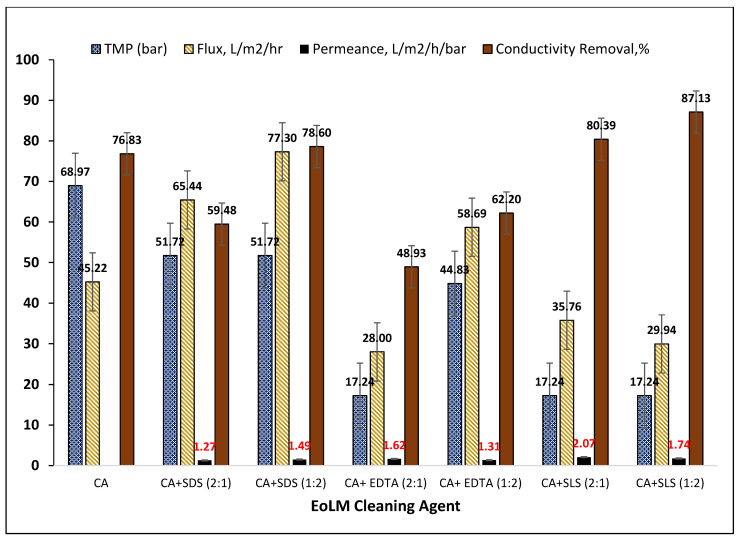
Performance of dialysate production EoLM cleaning using different chemical agents.

Figure 3 summarizes the key performance parameters for the REoLM originating from dialysis applications fouled by mixed organic, proteinaceous, and inorganic deposits and rehabilitated using various binary cleaning agents consisting of CA with surfactants (SDS, SLS) or chelator (EDTA). For CA alone, an applied TMP of 68.97 bar yielded a moderate flux of 45.22 L·m^−2^·h^−1^ and 76.83% conductivity removal. In comparison, combining CA with surfactants or chelating agents markedly improved cleaning efficiency and membrane recovery. Among surfactant-assisted treatments, CA + SLS (1:1) at 37.93 bar achieved the highest conductivity removal (90.20%) with a flux of 46.89 L·m^−2^·h^−1^ and good permeance (1.24 L·m^−2^·h^−1^·bar^−1^), suggesting enhanced surface hydrophilicity and restored selectivity. The highest flux overall (77.30 L·m^−2^·h^−1^) was observed with CA + SDS (1:2) at 51.72 bar, indicating SDS’s effectiveness in removing hydrophobic foulants. EDTA-based treatments primarily addressed inorganic scaling but were less consistent; CA + EDTA (1:1) produced relatively high flux (67.01 L·m^−2^·h^−1^); excessive EDTA (2:1) resulted in the lowest flux (28.00 L·m^−2^·h^−1^) and conductivity removal (48.93%). EDTA-based cleaning primarily targets inorganic scaling, thus yielding variable results. Overall, the data indicates that CA + SLS mixtures consistently outperform other combinations in the conductivity removal, particularly under lower TMP conditions, making them possessing higher energy efficient performance. SDS excels when maximum flux recovery is prioritized, while SLS offers the best balance of flux, permeance, and salt rejection. Comparable surfactant-assisted cleaning of fouled RO membranes has been reported by several researchers [19,21,22,23,24,25,26,27,28], summarized and compared with the present study’s results in Table 2. For instances, Ochando-Pulido et al. [25] achieved complete permeability restoration for olive mill wastewater RO membranes using 0.1% CA + NaOH + SDS at 2.7 bar, and Garcia-Fayos et al. [18] restored seawater RO membranes fouled mainly by silica/aluminum using 0.5% SDS at 40 °C, recovering permeate flux to 0.60 L m^−2^ h^−1^ bar^−1^ with a salt rejection index (SRI) of 96.8%.

Other similar works confirmed the importance of optimizing chemical type, concentration, and operating parameters. In this regard, Jung et al. [19] achieved ~72.4% flux recovery and >85% salt rejection by sequential acid (pH 3) and base (pH 12) cleaning at 45 °C for 3 h, using 0.5% EDTA and 0.01% SDS. Using RSM modeling, Park et al. [23] predicted ~86.6% flux recovery (FR) using 0.68 wt% EDTA at 20 °C, 20 min, and 409 mL min^−1^ flow rate, with an R^2^ of 83.95% for the regression model. Gul et al. [8] reported a remarkable 288.8% flux recovery for organically fouled nanofibrous PAN membranes using Triton + 5% NaOH, whereas excessive SDS during acidic cleaning caused a flux “stopper effect.” In a study reported by Li & Elimelech [26] similarly, they emphasized that alkaline + detergent cleaning outperforms acids alone for humic fouling. Collectively, these results confirm that surfactants are indispensable for organic fouling removal, but optimal performance depends on foulant composition, pH, chemical agents used and dosing strategies.

Studies on other different types of fouled RO polymeric membranes from wastewater treatment processes underscore context-specific cleaning needs. For instance, Masse et al. [22] found that for swine wastewater treatment RO membrane fouling, 18 mM SDS at pH 11/40 °C for 60 min or NaOH alone at pH 12/33 °C for 120 min gave the high FR after four cleaning cycles. Madaeni & Samieirad [21] reported that acids (HCl, HNO_3_, H_2_SO_4_) alone were ineffective, whereas a two-step NaOH–SDS followed by acid cleaning restored flux effectively for industrial wastewater fouled membranes. Generally, these studies suggest that acid–surfactant or alkaline–detergent sequences can restore 70–97% flux recovery ration (FRR) and >85–96% salt rejection when properly optimized for foulant and process conditions. The optimal cleaning protocol depends strongly on the membrane type, foulant composition, and intended reuse application. Acid–surfactant or acid–base strategies with controlled chemical doses are most effective for mixed organic–inorganic fouling and high-value reuse (e.g., dialysis), while alkaline-focused strategies suffice where inorganic or protein fouling dominates. The present study further advances the understanding of fouled RO membranes by demonstrating that CA–surfactant–chelator mixtures at moderate TMPs (17–69 bar) can achieve up to 90% conductivity removal and ~77 L m^−2^ h^−1^ flux, supporting sustainable dialysis wastewater reuse and aligning with circular economy goals [10]. Accordingly, Park et al. [23] further highlighted the value of statistical design-of-experiments (RSM/CCD) to optimize cleaning variables for achieving higher water flux recovery. Generally, these findings suggest that chemical dosing and operating conditions must be fine-tuned based on foulant type and membrane material. This demonstrates the need for optimizing the CA–surfactant cleaning for improved fouled membranes recovery performance to align with present study’s objectives of sustainable management of dialysate EoLM repurposing and reusing strategy.

### 3.2. Characterization of REoLM Membranes

#### 3.2.1. SEM and EDX Characterization in Relation to REoLM Performance

**Figure 4:**
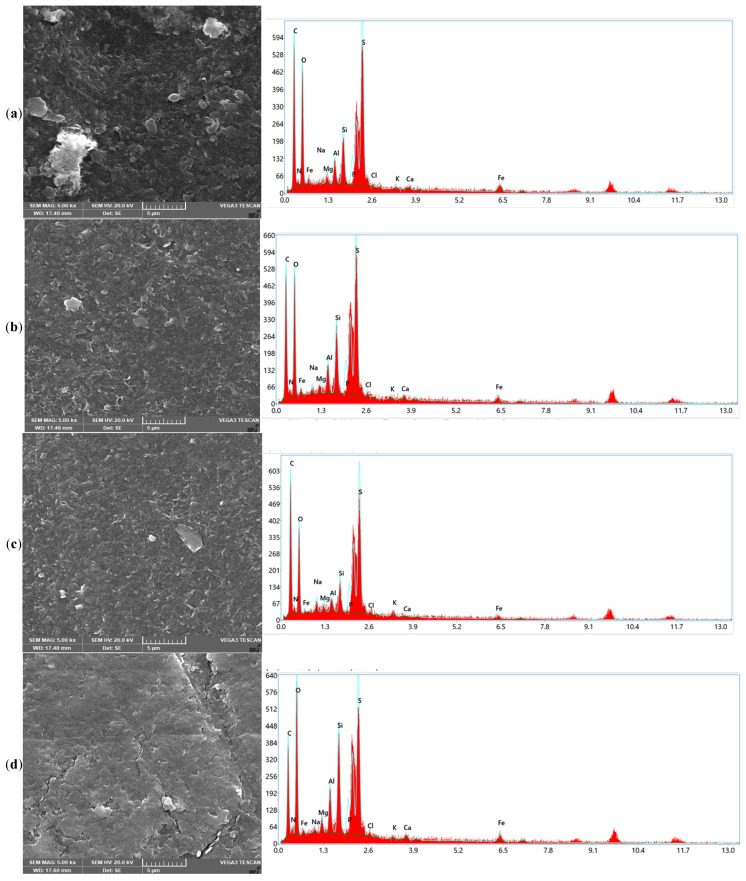

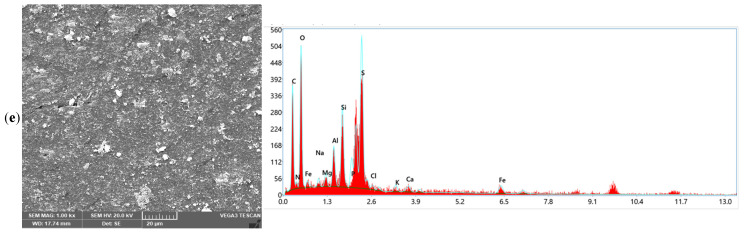
SEM images and EDX elemental distribution spectrum for the (**a**) fouled EoLM and REoLM cleaned using (**b**) CA, (**c**) CA + EDTA, (**d**) CA + SDS, and (**e**) CA + SLS.

The SEM and EDX analyses in Figure 4 provide complementary insights into the fouling characteristics of the end-of-life membranes and the mechanisms underlying their recovery after chemical cleaning. The SEM images in Figure 4 reveal clear distinct morphological transformations of the membrane surfaces after chemical cleaning that correlate with the performances of the different chemical agents depicted in Figure 1 for CA and in 50/50 binary combination matrixes with EDTA, SDS, and SLS, respectively. The fouled EoLM (Figure 4a) shows heavy deposition and irregular surface features, indicating a rough surface topography, characterized by dense aggregates, particulate deposits, and a heterogeneous layer likely composed of both organic and inorganic fouling that suppresses flux and ion rejection. This was attributed the additional fouling developed as results of damping storage conditions observed on physical inspection of the EoLM membrane, which likely compounded the fouling state of the original EoLM after removed from service. On the other hand, in contrast, all the REoLMs (Figure 4b–e) exhibit smoother and cleaner surfaces, especially those treated, suggesting effective removal of foulants. The darker regions are likely associated with the polyamide (PA) active layer, with sulfur (S) signals indicating residual sulfonate groups or cleaning agent residues [11], suggesting partial retention of the membrane’s original composition. The white regions, indicative of higher elemental density, predominantly feature silicon (Si) and aluminum (Al), with minor K and Na signals, consistent with alumino-silicate clay minerals such as kaolinite, likely deposited as inorganic foulants which are still partially retained after the cleaning. The gray regions reveal a heterogeneous mix of Na, Ca, Mg, Al, and Si suggesting surface deposition of inorganic impurities such as carbonate scales or silicates, reflecting the membrane’s exposure to hard water or residual contaminants during hemodialysis process. Thus, the EDX analysis corroborates SEM images’ revelation of distinct regional compositions on the rehabilitated membrane.

Considering the single CA-cleaned EoLM SEM image (Figure 4b), there is evidence of the partial removal of crystalline deposits, but it still depicts patchy organic layers, while the EDX confirmed residual carbon-rich deposits (C = 46.65%, O = 27.82%) with very low Ca and Fe signals. This can be attributed to modest flux recovery (45.22 L·m^−2^·h^−1^) and conductivity removal (76.83%) observed in the performance testing, demonstrating that CA chelation alone was insufficient to restore selectivity.

However, integrating the SEM and EDX results further elucidates the superior synergy and performance discrepancies for the binary CA–surfactant-based cleanings. The EoLM treated with CA + EDTA (Figure 4c) displayed smoother SEM surfaces where crystalline deposits had been stripped, and EDX confirmed the lowest Ca (0.28%) and Fe (1.41%) contents, validating lesser effective inorganic scale removal. However, the high residual carbon (46.65%) suggested persistent organic foulants, explaining the variable flux recovery observed: relatively high flux at a 1:1 ratio (67.01 L·m^−2^·h^−1^) but a sharp decline at 2:1 (28.00 L·m^−2^·h^−1^) with the poorest conductivity removal (48.93%). In comparison, CA + SDS cleaning resulted in SEM surfaces with fewer organic smears and clearer exposure of the polyamide skin layer (Figure 4d). EDX supported this, showing elevated O (34.81%) and N (4.61%) consistent with polyamide exposure, though silicate residues (Si = 5.06%, Al = 2.41%) remained. These changes corresponded with the highest water flux (77.3 L·m^−2^·h^−1^), indicating superior removal of hydrophobic organics but only moderate salt rejection. Meanwhile, Figure 4e shows the CA + SLS yielded SEM images of a uniformly cleaned and more hydrophilic surface, with EDX showing balanced O (33.91%) and N (3.64%) but lower silicate residues (Si = 4.08%, Al = 2.19%) compared to SDS. This correlated with the highest conductivity removal (90.20%) at moderate TMP (37.93 bar) and stable permeance (1.24 L·m^−2^·h^−1^·bar^−1^), indicating that SLS achieves the most balanced recovery of both flux and rejection. Collectively, these results demonstrate that while SDS promotes maximal hydraulic recovery by efficiently solubilizing organics, SLS achieves a more balanced outcome by enhancing both flux and rejection, making it the most effective cleaning reagent in terms of sustainable membrane repurposing.

#### 3.2.2. FTIR Characterization in Relation to REoLM Performance

**Figure 5:**
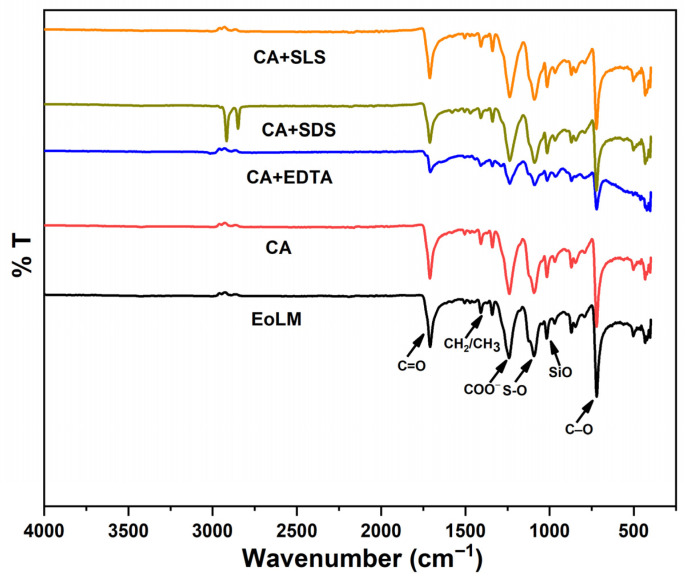
FTIR of EoLM and REoLM after cleaning using various agents.

The FTIR analysis spectrum depicted in Figure 5 provides critical insights into the chemical transformations on the surface of the fouled end-of-life membrane (EoLM) before cleaning. The fouled EoLM spectrum exhibits prominent absorption bands at ~3300 cm^−1^ (O-H stretch from polysaccharides/humic substances), ~2900 cm^−1^ (aliphatic C-H stretch from proteins/lipids), ~1700 cm^−1^ (C=O stretch from amide I or carboxylic acids), ~1400–1500 cm^−1^ (CH_3_/CH_2_/COO^−^ stretches from carboxylates), ~1100 cm^−1^ (S-O stretch from sulfonated organics or residual SLS), ~1050 cm^−1^ (Si-O) [8], ~717 cm^−1^ (C-O stretch from polysaccharides), ~800 cm^−1^ (CH_2_ rocking) [29], and ~700 cm^−1^ (β-glycosidic C-H bend from biofilms). These bands collectively indicate a hydrophobicity-inducing organic foulant layer (e.g., polysaccharides, proteins, lipids), which reduces membrane hydrophilicity and impairs baseline water flux and rejection performance. On the other hand, post-rehabilitation using CA shows moderate attenuation of these bands, suggesting partial organic removal and moderate performance enhancement. CA + EDTA further reduces COO and O-H intensities probably due to metal-chelated organic removal and yielding slightly better performance than using CA alone. CA + SDS suppresses protein-related C=O and S=O bands, resulting in variable performance gains. However, CA + SLS nearly eliminates all organic foulant peaks, restoring a virgin polyamide-like profile, which correlates with the overall superior performance among the tested agents at moderate TMP. The FTIR results corroborates excellent foulant removal which enhances hydrophilicity and permeability at lower TMP compared to the original EoLM, further reinforcing CA + SLS’s efficacy for sustainable membrane repurposing.

## 4. Modeling and Optimization of Creatinine Remediation Using REoLM

### 4.1. Development RSM Models for Rehabilitated EoL Hemodialysis Membranes

The model’s development for each of the four performance parameters for filtration tests for creatinine removal from water using the REoLM were achieved via fitting the obtained experimental data (Table 3) into the general polynomial RSM equation given in Equation (5) via regression approach with the aid of Design Expert version 8. Meanwhile, the quality of the developed models was evaluated and compared using coefficient of determination R^2^ and RMSE as per Equations (6) and (7), respectively [29]

(5) $$Y=\beta_{0}+\sum_{i=1}^{k}\beta_{i}x_{i}+\sum_{i=1}^{k}\beta_{i}x_{i}^{2}+\sum_{i=1}^{k-1}\sum_{j=2}^{k}\beta_{ij}x_{i}x_{j}+\varepsilon$$

where Y = dependent variable investigated; *β_ii_*, *β_ij_*, *β_i_*_,_ *β*_0_, are the developed model’s coefficients; *x_i_* and *x_j_*, = operational conditions.

(6) $$R^{2}=1-\frac{{\left(y_{i}-{\overset{´}{y}}_{i}\right)}^{2}}{{\left(y_{i}-\bar{y}\right)}^{2}}=1-\frac{SSR}{SST}$$

(7) $$RMSE=\frac{1}{n}\sum_{i=1}^{n}{\left(y_{i}-{\overset{´}{y}}_{i}\right)}^{2}=\frac{1}{n}\sum_{i=1}^{n}SST$$

where $y_{i}$ = actual value, $\bar{y}$ = mean value, ${\overset{´}{y}}_{i}$ = predicted value, *SSR* = residuals sum of squares, and *SST* = total sum of squares.

The developed models for the performance response variables for water flux, water permeance, and creatinine residual concentration and creatinine removal efficiency were designated as Y1, Y2, Y3, and Y4, given in Equations (8)–(11), respectively. The quality and predictive capabilities of these RSM models developed using FC-CCD for the performance of the citric acid/SLS-rehabilitated EoL RO membranes vary across the investigated response variables. Except for Y1, all the best fits for the other models required either squared or reciprocal transformations (square root for Y3 and reciprocal for Y2 and Y4) to ensure non-normality or to address heteroscedasticity while ensuring the best prediction accuracy is achieved, a common practice in RSM to improve model fit and interpretability.

Y1 = +37.32 − 2.65A + 2.70B + 19.57C + 2.19AB + 0.6501AC − 0.4137BC(8)

Y2 = +0.6832 + 0.0847A − 0.0833B + 0.0103C − 0.0815AB − 0.0612AC + 0.0584 + BC + 0.0527 + ABC (9)

Y3 = +44.86 − 0.0934A + 0.0025B + 0.0470C − 0.0220AB − 0.0743AC (10)

Y4 = +0.0124 − 0.0018A + 0.0003B + 0.0011C − 0.0006AB − 0.0016AC + 0.0005BC − 0.0006ABC(11)

The section below discusses the developed model fitting parameters, ANOVA analyses for the main effects, interaction effectiveness, and their contributions to the developed models’ quality.

### 4.2. Developed EoL Rehabilitation Models Diagnostics

**Figure 6:**
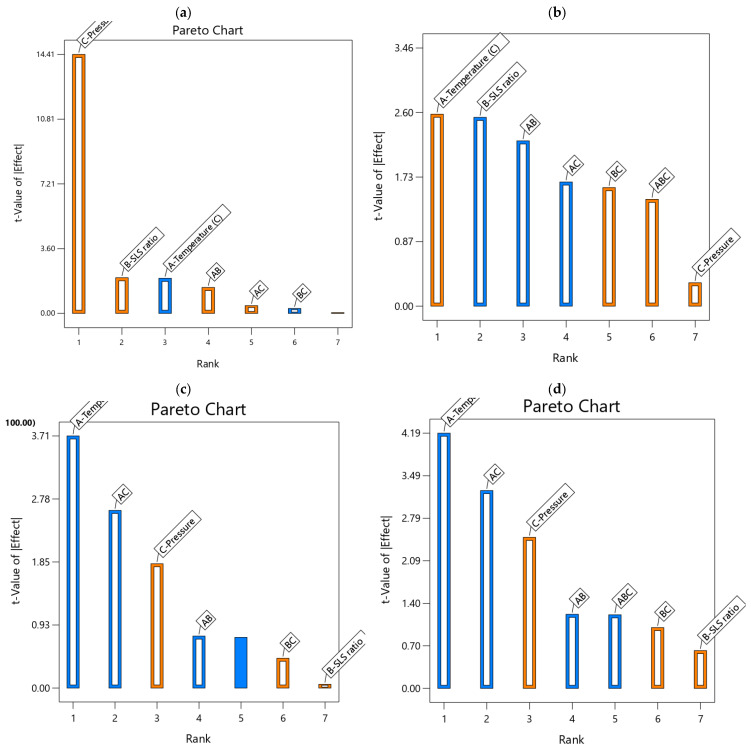
Pareto charts for (**a**) water flux, (**b**) water permeance, (**c**) creatinine residual concentration, (**d**) creatinine removal efficiency developed models (blue bars = antagonistic influence; orange bars = synergetic influence).

**Figure 7:**
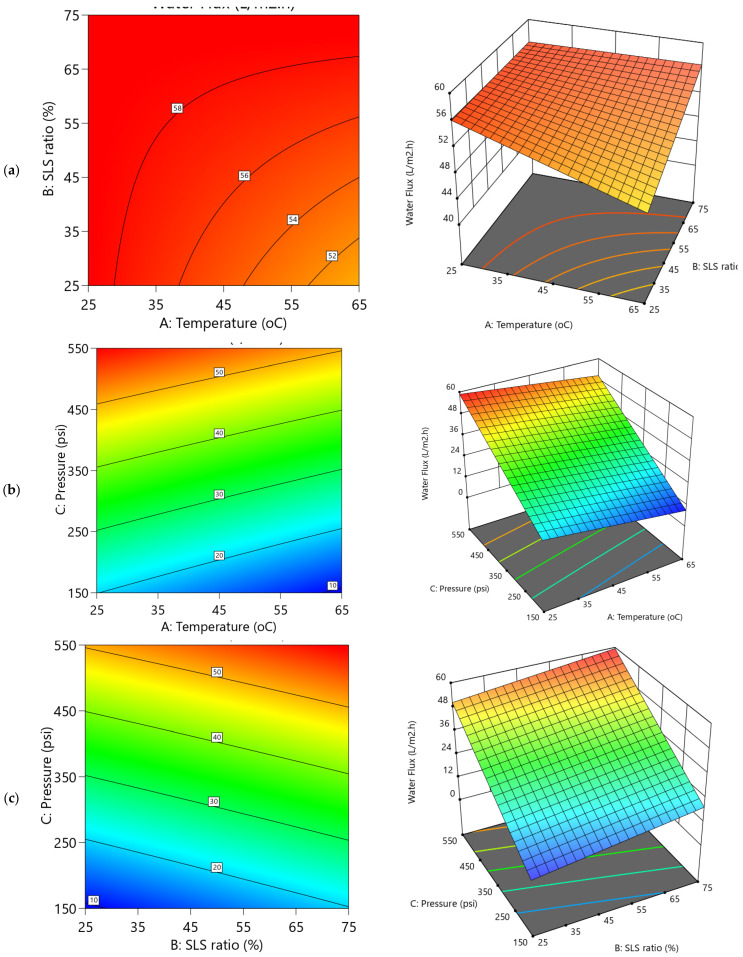
2D Contour plots and 3D diagrams for relationships of combined influence of operational conditions (**a**) Temperature vs. SLS ratio (**b**) Pressure vs. Temperature (**c**) SLS ratio vs. Pressure on water flux (Y1). The color gradient represents the magnitude of water flux (L/m^2^·h), increasing from blue (low) to red (high).

The developed models Y1 to Y4 were assessed and validated using data presented in Table 4 and Figure 6 and Figure 7. Accordingly, the models’ predictive abilities are also presented in respective plots in Figure S1 which compare observed and predicted values to assess model accuracy. In addition to R^2^ and RMSE, the quality of the models’ fittings were evaluated also using absolute error, Chi-Square and mean absolute percent error (MAPE, %) as provided in Table 4. The ranges of the R^2^ (0.714–0.96) and RMSE (0.0256–1.634) indicate high quality to moderately fitted models. For Y1 and Y4 (Figure S1a,d), a close alignment of the experimental data along the prediction that passes through the origin supports the obtained higher R^2^ (0.848 and 0.962) and lower RMSE (0.00255 and 1.634), thus exhibiting the strongest fit. However, for Y2 and Y3, the shattered plots (Figure S1b,c) reflect the reduced R^2^ of 0.775 and 0.818 with the latter showing more scattering comparatively yet exhibiting lower RMSE of 0.231 and 0.211438, also indicating good and consistent agreement of good model fitness. Similarly, all the other models’ quality check parameters that included absolute error, Chi-Square, and MAPE further supports all the models’ predictive potentials. To further reaffirm the models’ adequacies, the normal probability plots of residuals (Figure S2a–d) were assessed by evaluating normality assumptions, a key requirement for predictive models. As shown in Figure S2a, water flux (Y1) exhibits near-perfect alignment along the reference line, confirming normally distributed residuals—consistent with its high R^2^ (0.962) and non-significant lack of fit (*p* = 0.3075). Although minor deviations at extremes exist, which are negligible, thereby reinforcing the model robustness. For water permeance (Y2), residuals show slight S-shaped curvature, indicating mild non-normality. This aligns the moderate R^2^ (0.818) with the strongly desired insignificant lack of fit (*p* = 0.5357), which also suggest acceptable adequacy. Similarly, the Y4 residuals met the normality assumption as the normality line was followed closely, though with minor scatter at higher removal efficiencies (>95%), which could be attributed to the reciprocal transformation adopted that ensured the best fit of experimental data.

### 4.3. ANOVA and Impact of Main and Interactive Effects of REoLM Models

Tables S2 and S3 provide ANOVA and regression analyses, assessing main effects, interaction effectiveness, and their contributions to model quality. The regression coefficients in the equations represent the impact of each factor and their interactions with positive and negative coefficient indicating synergetic and antagonistic influence on the responses. Significant *p*-values (typically < 0.05) for main effects of A, B, and C would indicate that these factors individually influence the modeled responses. For instance, the negative linear terms in Y3 (−0.0934A) and Y4 (−0.018A) suggest that increasing citric acid concentration may reduce residuals or improve removal, consistent with its cleaning efficacy under low heat, while terms (0.025B, 0.0470C) indicate positive contributions from SLS and temperature. The inclusion of interaction terms (AB, AC) in the models indicates that the combined effect of factors is not merely additive. For instance, the −0.222AB in Y3 and −0.006AB in Y4 suggest that the joint effect of A and B may diminish the response, possibly due to competitive cleaning mechanisms between citric acid and SLS. The −0.743AC in Y3 and −0.016AC in Y4 highlight a strong antagonistic interaction between citric acid and temperature, potentially indicating optimal low heating limits. The interactions between negative coefficients indicate antagonistic effects, where combined factors reduce the response, thereby enhancing model complexity.

The ANOVA provided in Table S2 shows water flux (Y1) exhibited the strongest fit (R^2^ = 0.962, RMSE = 1.634), attributed to the higher influence of applied pressure (C), which contributed 87.98% to the model’s predictive power (*p* < 0.0001). Meanwhile, temperature (A) and SLS ratio (B) showed marginal significance (*p* ≈ 0.08), while there existed negligible interactions effects (AB, AC, BC) which could be attributed to the dominant impact of TMP. In contrast, the water permeance model (Y2) achieved a moderate fit (R^2^ = 0.818), with temperature (A) and SLS ratio (B) emerging as key factors (16.17% and 15.64% contributions, respectively; *p* < 0.035). The AB interaction (temperature vs. SLS) was influential (11.99%, *p* = 0.0575), suggesting synergistic effects on the improved membrane hydrophilicity due to impactive influence on removing fouling and the polyamide thin-film layer of the EoLM. However, pressure (C) was insignificant here, highlighting its divergent role in flux versus permeance in evaluating the REoLM performance. The significant curvature of Y1 and Y2 models (*p* = 0.0073 and 0.0105) further underscored the complexity of permeability parameters dynamics confirming non-linear behavior, validating the use of RSM quadratic function fitting. Water flux is known to be directly proportional to the applied pressure difference across the membrane, as described by the flux equation J_w_ = *k_w_*(ΔP−Δπ), which is evident in Y1. In contrast, in Y2, water permeance normalizes flux by the applied TMP, J_w_/ΔP. Accordingly, when pressure (C) is a dominant variable in the water flux model, its effect on permeance may be dwarfed or offset, especially if the membrane’s intrinsic permeability or fouling dynamics vary with pressure [2].

For the creatinine clearance-related responses’ ANOVA given in Table S3, creatinine residual (Y3) and removal efficiency (Y4) models showed moderate fits (R^2^ = 0.775–0.848) with temperature (A) as the most critical factor for both responses (33.92–35.21% contribution; *p* < 0.003). Pressure (C) significantly affected creatinine removal (Y4; 11.88%, *p* = 0.0382) but exhibited slightly lower impact on residual concentration at 10% confidence limit (Y3; *p* = 0.0777). Notably, the AC interaction was highly significant for both creatinine clearance models (17.84–20.37% contribution; *p* < 0.02), revealing synergistic integration of the distinct EoLM cleaning with the crossflow filtration process for the optimization of REoLM performance. However, SLS ratio (B) had minimal impact on the creatinine responses (*p* = 0.918 and 0.55, respectively).

The ANOVA results collectively emphasize that operational variables interact non-linearly to govern the REoLM performance. Pressure’s supremacy in flux (Y1) aligns with hydraulic-driven transport mechanisms, while temperature’s dominance in creatinine clearance responses (Y3/Y4) reflects thermodynamic control over removal of the fouled and polyamide EoLM surface which enhances the solute transport through the REoLM. The significant AC interactions for creatinine removal/residual (*p* < 0.02) indicate that simultaneous optimization of temperature and pressure is essential for maximizing rejection efficiency. Despite moderate fits for creatinine models, the low MAPE (0.13–7.22%) and Chi-Square values (<0.0001–0.0869) confirm predictive reliability. These models provide actionable insights: high-pressure operation (350–550 psi) optimizes flux, while moderate temperatures (45–65 °C) enhance creatinine removal.

### 4.4. Pareto Charts for Hierarchical Influence of the Operational Conditions

The Pareto Charts (Figure 6) rank the magnitude of the main effects and their interaction, helping to identify the most influential main factors and their interactions on the developed models’ performances. For the water flux (Figure 6a), applied pressure (19.57C) clearly dominates, contributing up to 87.98% of the model’s variance, with temperature (−2.65A) and SLS ratio (2.70B) having lesser but notable effects, reaffirming the results in Table 3. However, water permeance (Figure 6b) exhibited a balanced contribution from temperature (0.0847A) and SLS ratio (−0.0833B), each accounting to around 15–16% of the model’s variance, with interactions (e.g., −0.0815AB) adding to the complexity of the observed curvature. The charts highlight the temperature (−0.0934A) with a 35.21% contribution and AC interaction (−0.0743) at 17.84%; the creatinine residual concentration (Figure 6c) supports the data in Table 4, indicating their critical roles in reducing residuals. For creatinine removal efficiency (d), temperature (−0.0018A) contributes 33.92%, with AC interaction (−0.0016) at 20.37%, underscoring their impact on efficiency. These Pareto charts visually, further reaffirm the hierarchical influence of the operational conditions on the performance parameters, emphasizing the need to optimize pressure and temperature, with SLS ratio playing a supportive role, enhancing model interpretability.

## 5. Influence of Operational Conditions on REoLM Performance

### 5.1. Influence of Operational Conditions on Product Water Permeability

**Figure 8:**
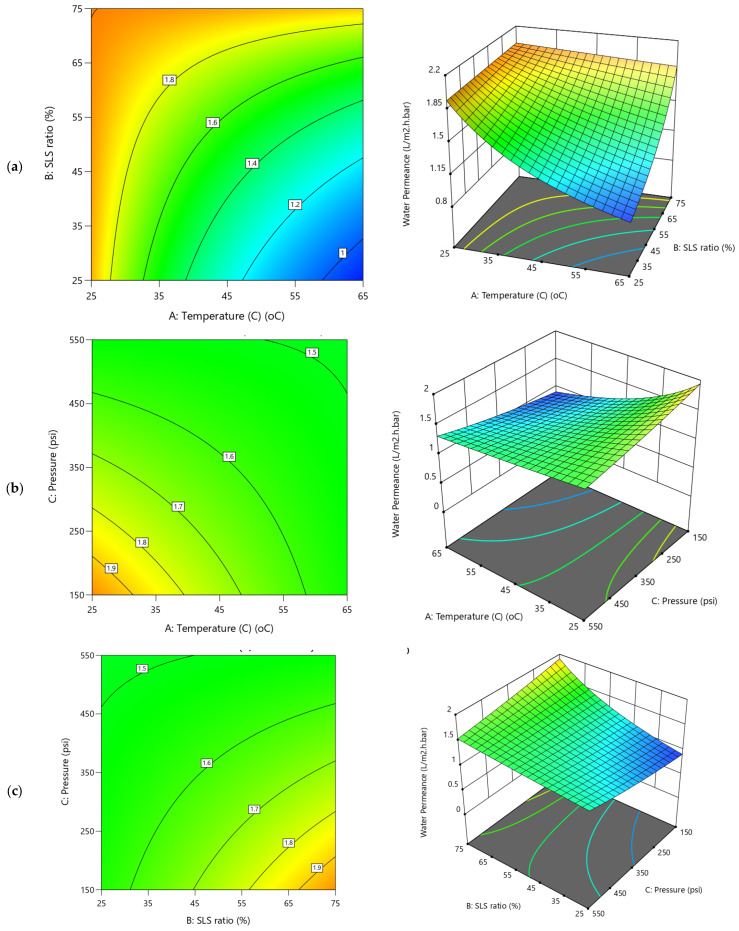
2D Contour plots and 3D diagrams for relationships of combined influence of operational conditions (**a**) Temperature vs. SLS ratio (**b**) Pressure vs. Temperature (**c**) SLS ratio vs. Pressure on water permeance. The color gradient represents the magnitude of water permeance (L/m^2^·h.bar), increasing from blue (low) to red (high).

**Figure 9:**
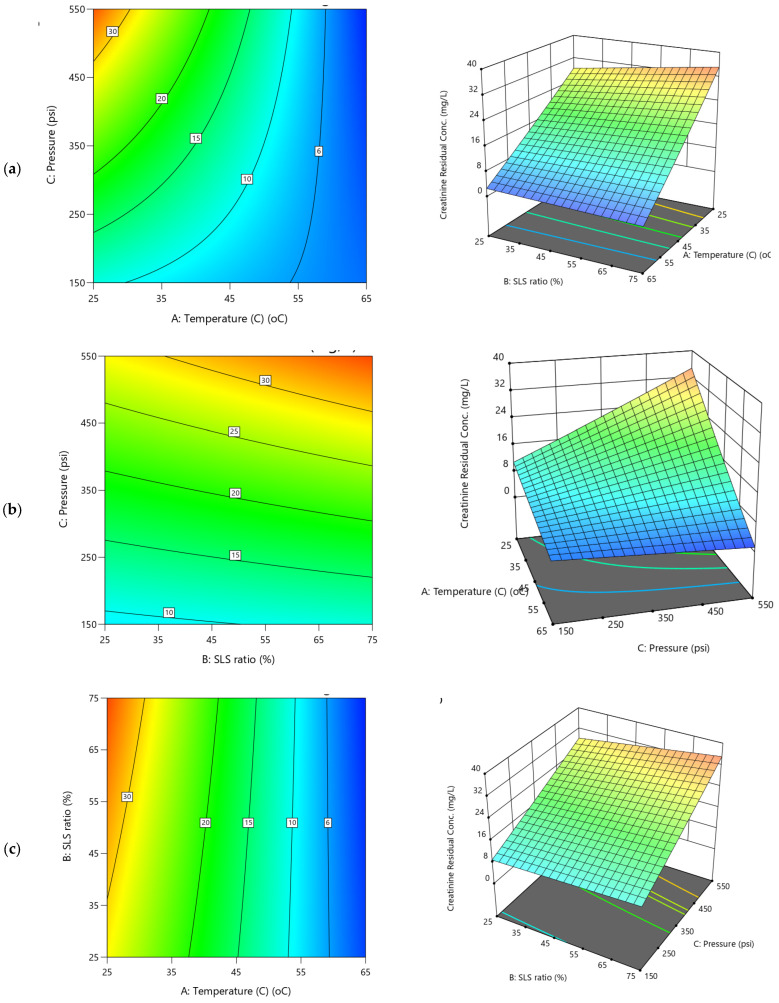
2D Contour plots and 3D diagrams for relationships of combined influence of operational conditions (**a**) Temperature vs. SLS ratio (**b**) Pressure vs. Temperature (**c**) SLS ratio vs. Pressure on creatinine residual concentration (Y3). The color gradient represents the magnitude of creatinine residual (mg/l), increasing from blue (low) to red (high).

**Figure 10:**
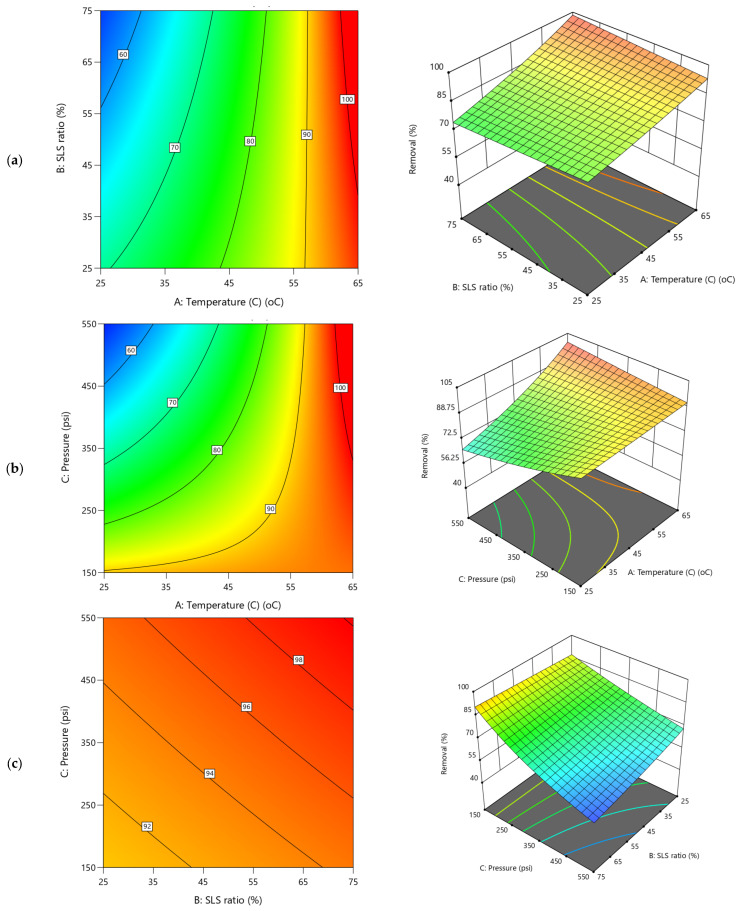
2D Contour plots and 3D diagrams for relationships of combined influence of operational conditions (**a**) Temperature vs. SLS ratio (**b**) Pressure vs. Temperature (**c**) SLS ratio vs. Pressure on creatinine removal efficiency (Y4). The color gradient represents the magnitude of creatinine removal (%), increasing from blue (low) to red (high).

The dependencies of water permeability parameters on operational conditions are depicted in the 3D and their respective contour plots (Figure 7 and Figure 8). The variability of water flux (Figure 7b,c) reveals a pronounced upward trend with increasing applied transmembrane pressure (TMP), exhibiting steep inclines that indicate a strong positive correlation. This is consistent with the statistical analysis of the Y1 model (Table S2), where TMP exhibits the dominant effect (coefficient 19.57C) contributing to approximately 87.98% of the variation in the water flux. Accordingly, increasing the pressure from 150 to 550 psi markedly enhanced flux from 17.97 to 58.63 L·m^−2^·h^−1^, demonstrating TMP’s predominant role in driving water through the membrane. In contrast, temperature and SLS ratio showed more subdued effects due to the predominant role of TMP (Figure 7a–c). The temperature exhibited a slight decline as it increases to higher levels (Figure 7a,b), couple with coefficient –2.65A in Table S2, suggesting a potential reduction in flux as temperature rises. Meanwhile, the SLS ratio (Figure 7a,c) exerted a comparative positive influence (2.70B; ~5.4% contribution), gradually enhancing flux with higher surfactant proportions to CA. These trends are visually reflected in the contour plots in Figure 9 and Figure 10, which display tightly packed elliptical curves along the pressure axis—indicating rapid flux variation with pressure—whereas contours along the temperature and SLS axes are more widely spaced, confirming their weaker impact. The REoLM, exhibiting a water flux of 59.36 L/m^2^·h and a permeance of 2.87 L/m^2^·h·bar, retains approximately 80–90% of the hydraulic performance of a new polyamide TFC membrane. This indicates effective restoration of permeability and justifies its reuse in ultrapure or secondary treatment applications, depending on salt rejection performance.

For water permeance (Figure 8), the 3D diagrams and corresponding contour plots show a more balanced influence across operational variables. Unlike flux, pressure exerts only a marginal effect (Figure 8b,c and coefficients in Table S2), while the temperature positively influence the permeance (Figure 8a,b), the SLS ratio (Figure 8a,c), had a negative influence, although both at ~15–16% contributions (Table S2). This suggests that higher temperatures (e.g., 65 °C) improve membrane flexibility and fouling removal, modestly boosting permeance, whereas increasing SLS ratios beyond mid-values may leave residual surfactants that reduce permeability. Notably, the negative AB (–0.0815) and AC (–0.0612) interactions indicate antagonistic effects when temperature and SLS or temperature and pressure increase simultaneously, consistent with the significant curvature term (0.2185; 26.93%) of the Y2 model. These distinctive behaviors between water flux and water permeance would be possibly due to dwindling dominant contributions of the applied TMP on the water permeance which could be attributed to increased fouling resistance. Thus, the irregular and overlapping contour patterns further underscore the complex interactions between these filtration variables and highlight the need for multi-factor optimization.

The forgone observations are in agreement with Park et al. [23], that reported optimized chemical cleaning for organic-fouled RO membranes using RSM and reported significant interaction and curvature effects. Their models achieved R^2^ of ~84% and a predicted flux recovery of 86.6% under optimized conditions (0.68 wt% EDTA, 20 °C, 20 min, 409 mL min^−1^). Additionally, in a different reported study, Jung et al. [19] optimized the cleaning of discarded RO membranes and observed hydraulic driving force as one of the primary determinants of flux recovery and salt rejection. Their optimal protocol for both acid and base cleaning were for 3 h at 45–50 °C, resulting in ~71–77% flux recovery with >80% salt rejection and further improvement to 85% flux recovery. Their trends align with the present findings that TMP and temperature have the greatest influence on flux, while permeance is strongly affected by temperature–chemical synergies and non-linear interactions (Figure 7a–c). Their results also emphasized that hydraulic driving forces dominate flux recovery, while permeance is more sensitive to temperature–chemical synergies and curvature effects, corroborating the trends observed here. Taken together, both studies demonstrate that while TMP is the primary determinant for flux, optimized recovery of permeance and selectivity requires careful balancing of temperature and chemical ratios to mitigate antagonistic interactions. The use of RSM and non-linear modeling is therefore, critical for accurately capturing these dependencies and for developing energy-efficient cleaning protocols for EoL membranes.

### 5.2. Influence of Operational Conditions on Remediation Creatinine from Water

The influence of the operational conditions investigated on creatinine clearance parameters are presented in the 3D and contour plots presented in Figure 9 and Figure 10, respectively. Despite their interrelationships, the models for the two responses (Y3 and Y4) exhibit significant divergent behaviors which were attributed to the significant impact of the variability of the operational parameters. The 3D diagrams for creatinine residual concentration display a clear downward trend with increasing temperature (Figure 9a,b), indicating a significant reduction in residual concentration as temperature rises. Conversely, the applied TMP shows a steep upward trend for the entire SLS ratio and especially at lower temperatures, suggesting a significant positive impact that resulted increase in residuals at higher pressure levels. The SLS ratio impact trend is nearly flat (Figure 9a,c and coefficient 0.0025B in Table S3), with minimal surface variation, indicating a negligible direct effect on the creatinine residual. These observations are clearly depicted in the strong negative effect of temperature (−0.0934A, 35.21% contribution in Table S3) on creatinine residual concentration, reducing residuals at higher levels (e.g., 65 °C), which was attributed to enhanced cleaning with CA and SLS. Although, TMP impact (0.0470C, 8.91%) shows a positive yet its exhibited lesser effect (Figure 9b,c). The negative AC (−0.0743, 17.84%) interaction suggests that combining temperature and pressure reduces residuals more effectively. Higher TMP increases the water permeation, yet as detrimental of increasing the residual creatinine in the filtered water driven by more convective creatinine transport across the membrane which were susceptible to curtail additional clearance. The contour plots feature steep, narrow bands along the temperature axis (Figure 9a), reflecting sharp changes in residuals, while the pressure and SLS ratio axes show broader (Figure 9a,c), more diffuse contours, underscoring their comparative weaker influence on the creatinine residuals.

Meanwhile, in the case of creatinine removal efficiency, Y4 (Figure 10), substantial improvement was evident as both temperature and TMP increase (Figure 10a,b) supporting the Pareto charts (Figure 6d). The applied pressure exhibiting a gentle upward slope when interacted with temperature (Figure 10b) which indicates a modest impact on enhancement in removal efficiency with higher pressure. Conversely the TMP impact is more significant when interacted with SLS ratio (Figure 10c). The SLS ratio impact on Y4 trend is either nearly flat or slightly upward, implying a limited direct impact on removal efficiency (Figure 10a,c). This is corroborated by the significant negative effect of temperature (−0.0018A, 33.92% contribution, Table S3) on removal efficiency, improving the efficiency of up to 99.9% at higher levels (e.g., 45–65 °C) due to effectiveness of the REoLM in the creatinine removal. The polyamide layer acts as a barrier layer and restricts the passage of creatinine molecules through the membrane. Based on the results, it is speculated that the rejection mechanism of creatinine is mainly governed by multiple interactions mainly involving size exclusion and electrostatic repulsion due to small molecular size and low charge density, respectively [2].

Moreover, the ANOVA given in Table S3, shows that the TMP effect (0.0011C, 11.88% contribution) and SLS ratio (0.0003B, 0.742%) having positive impact with the SLS ratio main effect exhibiting insignificant effects on the residual removal efficiency (*p*-value = 0.5527). The negative AC (−0.0016, 20.37%) and AB (−0.0006) interactions suggest that combined increases in temperature and pressure, or temperature and SLS, enhance removal efficiency, likely due to their combined synergistic effectives. Across all 3D and contours plots (Figure 10a–c), there is consistent demonstration of temperature as the dominant main effect parameters, with pronounced slopes in both upward (for removal efficiency and permeance) and downward (for residual concentration) directions. The second most significant factor was the TMP, suggesting the critical role of the temperature (33.9% and 35.2%) and TMP (11.88 and 8.91%) in influence the REoLM creatinine removal performance. Meanwhile, applied pressure mainly shows a strong positive trend dominating water flux but with a smaller influence on other responses, while temperature and SLS ratio balance water permeance. Interaction effects (e.g., AC, AB) add the curvature and non-linear complexity, validated by diagnostic analysis, enhancing model reliability. Generally, these insights provide the needs for optimization of rehabilitation conditions for sustainable wastewater treatment. The contour plots reinforce these trends with varying density and shape, with tighter contours reflecting rapid response changes (e.g., pressure on flux, temperature on residuals) and broader contours indicating gradual shifts (e.g., SLS ratio effects), providing a visual representation of the operational conditions’ differential impacts on performance of the REoLM on creatinine removal from water.

## 6. Numeral Optimization and Desirability Analysis for Sustainable Performance

### 6.1. Optimization Desirability Function Analysis

The RSM models successfully quantified the effects of operational variables on rehabilitated EoL membrane performance. Pressure and temperature emerged as dominant factors for flux and creatinine removal, respectively, while SLS ratio primarily influenced permeance. The significant interactions between the studied responses highlight the need to optimize the operational conditions to ensure balanced treatment process. Accordingly, numerical optimization of the REoLM for wastewater treatment reuse was conducted and interpreted using the desirability function capabilities of the Design Expert^®^ 8 software considering six (6) optimization scenarios given in Table S4 This approach provides a robust framework for balancing multiple performance indicators under varying operational conditions. Design Expert^®^ 8 uses specific desirability functions to calculate individual desirability scores (*d_i_*) depending on a maximization or minimization goal using Equations (12) and (13) respectively.

(12) $$d_{i}={\left(\frac{Y-Y_{min}}{Y_{max}-Y_{min}}\right)}^{s}$$

(13) $$d_{i}={\left(\frac{Y_{max}-Y_{min}}{Y_{max}-Y_{min}}\right)}^{s}$$

where *Y* is the predicted response, *Y_min_* and *Y_max_* are the lower and upper limits, and *s* is a weight (typically 1 unless adjusted) that controls the shape of the function. A higher *Y* closer to *Y_max_* or lower *Y* closer to *Y_min_* increases di toward 1, respectively. Meanwhile, for a specific optimization scenario, the composite desirability *D* is calculated as the geometric mean of the individual desirability scores:

$$D=({d_{1\;}.d_{2}.d_{3}.d_{4}\ldots \ldots .d_{n})}^{1/n}$$

where *n* is the number of responses. This ensures that *D* ranges from minimum = 0 (if any *d_i_* = 0) to maximum= 1 (if all *d_i_* = 1).

This implies that, the desirability function transforms each response variable into an individual desirability score (*d_i_*), which ranges from 0 (completely undesirable) to 1 (fully desirable) based on the target goal for that response (e.g., maximize, minimize, or target value) [30]. The specific individual desirability scores are then combined into a composite desirability (*D*), which serves as the overall optimization criterion. The software iteratively adjusts the input variables within their specified ranges or target constraints to maximize *D*, thereby identifying the optimal operating conditions. Thus, the combined desirability scores *D*, reflecting the overall success in achieving these combined goals offers a valuable insight into the optimal rehabilitation strategy for sustainable water treatment applications. For this present study, the desirability approach numerical of optimization capabilities were employed via Table S4 which presents six (6) scenarios, each covering combined, divergent target optimization objectives as goals for both EoLM rehabilitation operational conditions and performance indicators.

### 6.2. Optimization Results for EoLM Creatinine Filtration Performance

**Figure 11:**
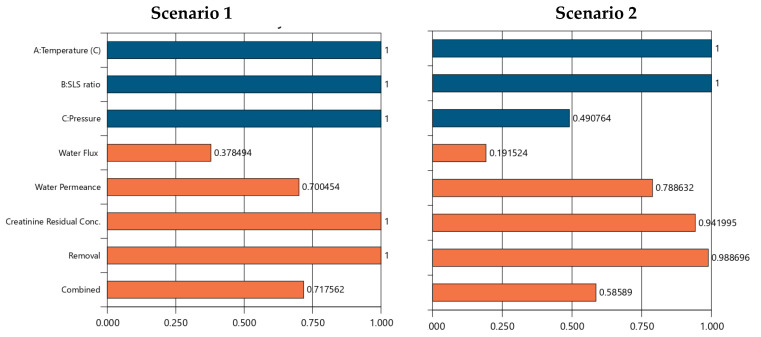

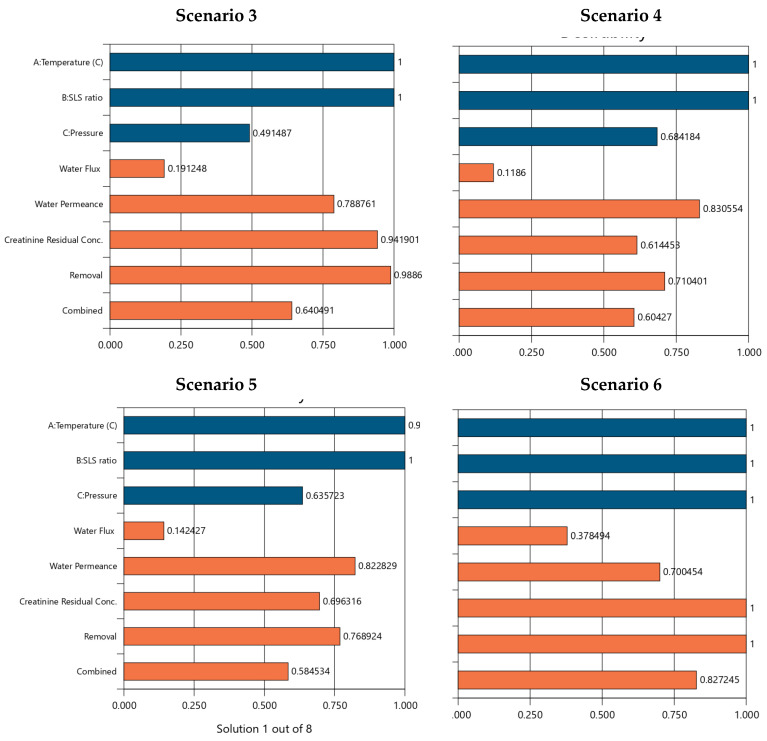
Scenarios 1 to 6 numerical optimization Desirability (*D*) results for REoLM creatinine removal from water. Blue color: target operational conditions goals achievements and orange color: target performance goals achievements as per Table S4.

The numerical optimization results for the scenarios 1–6 are presented in Table S4 and Figure 11. The outcome demonstrates Scenario 6 which maximizes all variables (temperature at 65 °C, SLS ratio at 75%, pressure at 550 psi), achieving the highest desirability score of 0.827, mirroring Scenario 1’s performance outcomes (water flux: 59.363 L/m^2^/hr, water permeance: 1.545 L/m^2^/hr/bar, zero residual, 100% removal efficiency). This reinforces the synergy of high temperature, SLS ratio, and pressure as an optimal operating region, achieving peak performance across all responses. Thus, scenario 6 is achievable to the detriment of increased energy demands to sustain the higher temperature and pressure requirements which are not desirable for reuse of EoL operations as it offers a less promising strategy for achieving sustainable water reuse management. CA + SLS cleaning temperature emerges as a dominant factor influencing REoLM performance, maximizing it across Scenarios 1, 2, 3, 5, and 6, consistently correlating with higher water flux, improved removal efficiency, and reduced or zero creatinine residuals, suggesting optimal cleaning efficiency under elevated conditions. In contrast, minimizing temperature (25 °C in Scenario 4) leads to the poorest removal and highest residuals, indicating a threshold below which chemical rehabilitation efficacy diminishes. The SLS ratio exhibits a threshold effect, with higher levels (75% in Scenarios 1–3, 6) enhancing permeance and removal, while minimization (25% in Scenario 4) or optimization (40.712% in Scenario 5) reduces performance, likely due to insufficient surfactant action or over-dilution. Meanwhile, applied pressure shows a strong positive trend for water flux at maximum levels (550 psi in Scenarios 1, 6), but minimization (276.32–412.85 psi in Scenarios 2–5) lowers flux while potentially improving permeance, indicating a trade-off between flow rate and permeability. The trends underscore the critical role of synergy of EoL cleaning temperature and the crossflow-applied TMP during the remediation of the creatinine-laden wastewater, with SLS ratio providing supportive enhancement. While the optimization provides an optimal scenario, the narrow range of desirability scores (0.566–0.827) and the complexity of the process indicates that trade-offs are inevitable when prioritizing specific responses for attaining sustainable EoLM rehabilitation and reuse.

Despite the compounded fouling state of the tested EoLM, the rehabilitation process was very effective in yielding high removal of salt and creatinine- with potential of the REoLM regaining up to 80% of the hydraulic performance of new polyamide TFC RO membrane. This superior REoLM performance aligns with global sustainability goals by repurposing EoLMs, reducing landfill disposal option, and offering a cost-effective alternative to management of dialysate EoLM. The optimization results suggest potential scalability for dialysis centers and healthcare waste management sectors, where optimizing spent dialysate wastewater treatment could be achieved. Moreover, this study comparatively demonstrates the potential of using environmentally friendly and more economical and chemical agents at lower concentration to achieve this purpose compared to other methods [15,16].

## 7. Conclusions

This study demonstrates a sustainable and cost-effective strategy for rehabilitating end-of-life (EoL) reverse osmosis (RO) membranes from hemodialysis centers using optimized low-temperature chemical cleaning and operational control. Using different cleaning agents that included citric acid (CA), EDTA, sodium lauryl sulfate (SLS), and sodium dodecyl sulfate (SDS), the mixture of CA and SLS (1:1) was found to be the most effective for balanced flux recovery, salt rejection, and creatinine clearance at lower TMP, achieving 90% conductivity reduction, 46.89 L/m^2^/h water flux, and 1.24 L/m^2^/h/bar permeance. A combined cleaning protocol with CA and SLS was further evaluated for its ability to restore membrane performance and enhance creatinine -a model spent hemodialysis contaminate- removal from water. Using response surface methodology (RSM) key operational variables were optimized for creatinine removal from water. Developed RSM models (R^2^ 0.775–0.962) yielded good predictions of water flux, permeance, creatinine residual concentration, and removal efficiency, with pressure being the dominant factor for flux and temperature influencing creatinine rejection. Under optimal conditions (65 °C, 75% SLS, 550 psi), water flux reached up to 59.36 L/m^2^·h with a permeance of 2.87 L/m^2^·h·bar, while achieving > 98% creatinine removal efficiency (desirability 0.827). Despite the compounded fouling state of the tested EoLM, after the rehabilitation process, the REoLM was very effective in removal of salt and creatinine- with potential of the regaining up to 80% of the hydraulic performance of the new polyamide TFC membrane. This indicates effective restoration of permeability, justifying its potential for reuse in ultrapure or secondary treatment applications. Characterization via FTIR and SEM/EDX, analysis confirmed both organic and inorganic fouling removal and improved surface hydrophilicity post-rehabilitation. These findings suggest that the rehabilitation process has potential of reducing solid waste, extend polymeric TCF membrane lifespan and lowers dialysate production operational costs. Future work should focus on pilot-scale validation with real spent dialysate, long-term stability assessments, and integrated economic and lifecycle analyses to fully realize its environmental and economic benefits to open a pathway toward closed-loop water management and circular economy practices at dialysis centers.

## Figures and Tables

**Table 1:** Actual and coded values for soil washing operational conditions.

Variable	Unit	Variables (Coded) Values		
Independent (operational) values and levels				
		Low (−1)	Mid (0)	Low(+1)
A: Temperature	°C	25	4.5	65
B: SLS ratio	%	25	50	75
C: Applied Pressure	psi	150	350	550

**Table 2:** Comparative chemical agents cleaning studies.

Membrane Type	Fouling Type	Cleaning Agents	Operational Condition and Performance	Reference
TFN RO (BW440-ES, LG)	Organic (humic acids) + Inorganic (salts, gypsum)	Citric acid (0.2%) + EDTA·4Na (0.1%) (CA → EDTA vs. EDTA → CA)	Filtration 12 bar, 24 h, 5–45 °C; fouling thickness with temperature (4.03–6.54 µm); FRR > 95% when citric acid used first; FRR declined to ~90% after 3 cycles; CE sequence (CA first) more effective at foulant removal	[24]
TFC Polyamide RO (spiral wound)	Mixed organic, protein, inorganic	CA, CA + (SDS, SLS or EDTA binary)	CA alone: 45.2 LMH, 76.8% cond. removal @ 68.97 bar; CA + SLS: 46.9 LMH, 90.2% cond. removal, 1.24 LMH/bar @ 37.93 bar; CA + SDS: 77.3 LMH @ 51.72 bar; EDTA (2:1) lowest flux 28 LMH, 48.9% cond. removal	Present study (Dialysis EoL RO)
TFC-1 RO (Hydranautics)	Organic (alginate, SRNOM) in the presence of Ca^2+^	NaOH (pH 11), EDTA (0.5–2 mM, pH 11), SDS (2–10 mM, pH 11)	NaOH ineffective (<15% recovery); EDTA efficiency increased with dose and pH (up to ~100% at 2 mM, pH 11, 40 °C, 60 min); SDS effective only above CMC (~8 mM), nearly 100% recovery at 10 mM, pH 11, 60 min;	[27]
TFC RO (LFC-1, Hydranautics)	Mixture of organic foulants alginate, BSA, SRNOM, and octanoic acid) with Ca^2+^	SDS, NaCl and EDTA (pH 11)	Effective foulant removal and flux restoration (>90% FRR). 15 bar	[28]
TFC-PA RO	Organic + Inorganic	NaOH (pH 11), HCl (pH 2),	45 °C, 3 h; 65–75% → >85% @ 15 bar	[28]
Hydrophilic RO membrane oily wastewater treated	Organic + inorganic	Citric acid + NaOH + SDS (2-step)	Complete permeability restoration; 0.1% CA + NaOH + SDS; 2.7 bar; 30–35 °C; 20–25 min	[25]
Seawater RO membrane	Mainly colloidal/inorganic (silica, NOM)	SDS 0.5% @ 40 °C; NaOH alt.	Permeate flux: 0.60 L/m^2^·h·bar; SRI 96.8%; SDS 0.5% @ 40 °C, 1 h static cleaning	[18]
RO membrane (type not specified)	Organic fouling (humics, NOM)	NaOH + detergent	Alkaline–detergent > acids; effective organic cake removal; no specific flux given	[26]
Nanofibrous PAN membrane	Organic/protein	SDS, NaOH, Triton (varied conc.)	Flux recovery: up to 288.8%; Optimal 1 wt% NaOH + Triton; Excess SDS in acidic cleaning caused flux stopper effect	[8]
Discarded polyamide RO membrane	Mixed foulants	Acid (pH 3) + base (pH 12) + EDTA/SDS	Flux recovery: ~72.4%; Salt rejection > 85%; 0.5% EDTA, 0.01% SDS; 45 °C; 3 h	[19]
RO membrane (organic fouling)	Organic fouling	EDTA 0.68% (optimized via DoE)	FR ~86.6% predicted; R^2^ = 83.95%; 0.68 wt% EDTA; 20 °C; 20 min; flow rate 409 mL/min	[23]
	Protein/inorganic (swine wastewater)	SDS-NaOH (pH 11–12), EDTA (varied)	FR highest with 18 mM SDS @ pH 11/40 °C (60 min); NaOH @ pH 12/33 °C (120 min); 4 cycles	[22]
FT-30 hydrophilic polyamide membrane	Organic + inorganic (industrial waste)	NaOH + SDS followed by acid	Two-stage NaOH–SDS then acid; fouled 540 min; high FR; acids alone ineffective	[21]

**Table 3:** Experimental results for performance of REoLM remediation of creatinine from water.

Standard	Run Order	Operational Conditions			Performance Indicators			
Design								
Order								
		A: Temperature, °C	B: SLS Ratio,	C: Applied Pressure,	Y1: Water Flux, L/m^2^/hr	Y2: Water Permeance, L/m^2^/h/bar	Y3: Creatinine Residual, mg/L	Y4: Creatinine Removal,
			%	psi				%
11	R1	45	25	350	32.15	1.29	19.43	75.71
4	R2	65	75	150	17.97	1.68	2.66	96.68
13	R3	45	50	150	23.17	2.16	16	80
16	R4	45	50	350	29.79	1.19	0.08	99.89
8	R5	65	75	550	58.63	1.49	1.53	98.09
1	R6	25	25	150	17.97	1.68	5.69	92.89
14	R7	45	50	550	58.63	1.49	3.47	95.66
5	R8	25	25	550	57.68	1.47	26.45	66.94
17	R9	45	50	350	24.59	0.98	0.08	99.89
9	R10	25	50	350	45.39	1.82	26.48	66.91
3	R11	25	75	150	17.97	1.68	4.48	94.41
10	R12	65	50	350	30.73	1.23	2.97	96.29
2	R13	65	25	150	9.46	0.88	4.04	94.94
7	R14	25	75	550	55.79	1.42	37.82	52.73
15	R15	45	50	350	29.31	1.17	0.08	99.89
12	R16	45	75	350	45.39	1.82	17.31	78.37
6	R17	45	25	550	51.54	1.31	5.85	92.69

**Table 4:** Model regression fits parameters.

Parameter	Y1: Water Flux	Y2: Water Permeance	Y3: Creatinine Residual	Y4: Creatinine Removal
R^2^	0.962	0.818	0.775	0.848
Absolute error	2.669	0.054	0.044706	0.000653
RMSE	1.634	0.231	0.211438	0.025553
Chi-Square	13.040	0.184	0.0869	0.000077
MAPE (%)	8.290	6.280	0.13	7.22

## Data Availability

Data is contained within the article or Supplementary Materials.

## Supplementary Materials

The following are available online at https://www.mdpi.com/article/10.3390/polym17212922/s1, Figure S1: Experimental vs developed models predicted plots for (a) Water flux (d) water permeance (c) creatinine residual concentration (d) creatinine removal efficiency developed models; Figure S2: Normal probability plots for (a) Water flux (d) water permeance (c) creatinine residual concentration (d) creatinine removal efficiency developed models; TableS1: Manufacturer’s specifications of the pristine dialysate center RO membranes (FilmTec™ HSRO-4040-FF Element.); Table S2: RSM models ANOVA for REoLM water flux and permeance; Table S3: RSM models ANOVA for REoLM creatinine residual concentration and removal efficiency; Table S4. Rehabilitated EoL membrane performance numeral optimization goals, results, and desirability for the different target scenarios.
