# Supplementary material for: Repurposing of End-of-Life Dialysate Production Polymeric Membrane for Achieving Sustainable Hemodialysis Process Water Management
Source: Polymers (Basel). 2025 Oct 31;17(21):2922. doi: 10.3390/polym17212922 (PMC12608294; doi:10.3390/polym17212922)
Supplement: Supplementary file 1 [file polymers-17-02922-s001.zip › polymers-3906422-supplementary.pdf]

<u>Supplementary Materials</u>

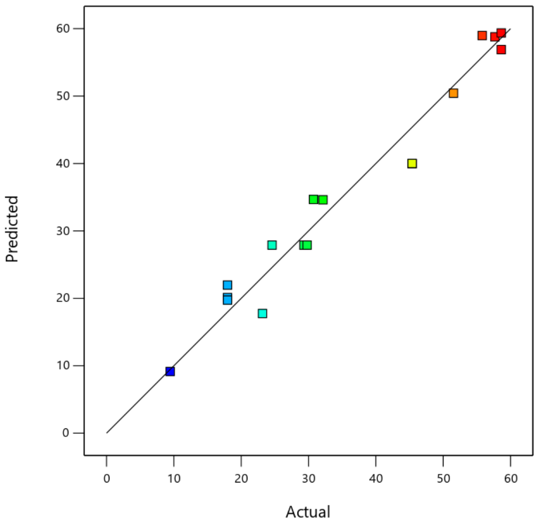

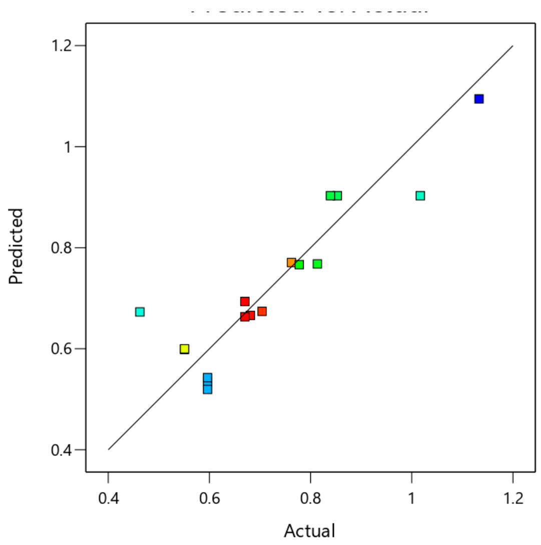

(C) **(d)**

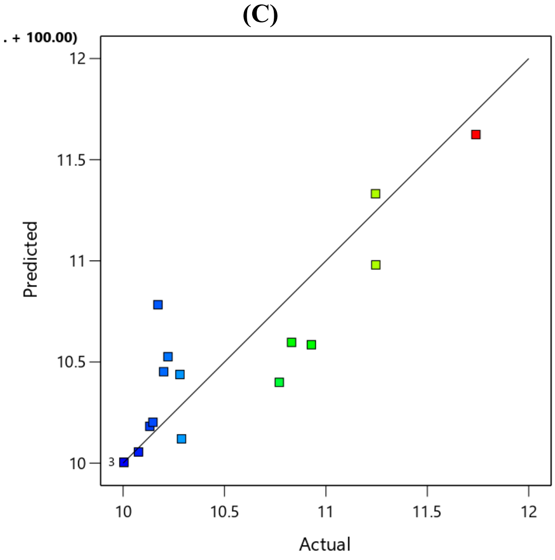

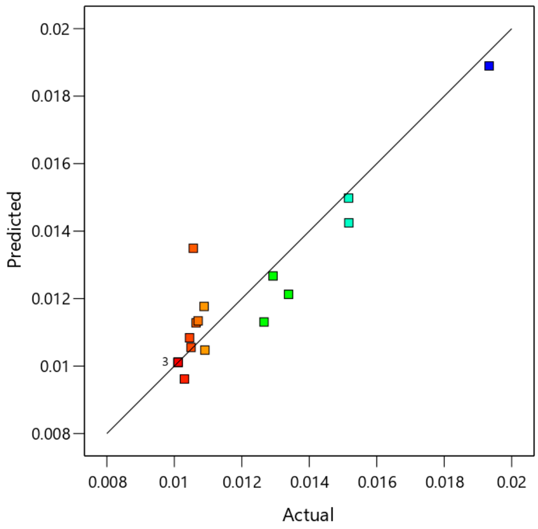

Figure S1: Experimental vs developed models predicted plots for (a) Water flux (d) water permeance (c) creatinine residual concentration (d) creatinine removal efficiency developed models

(b)

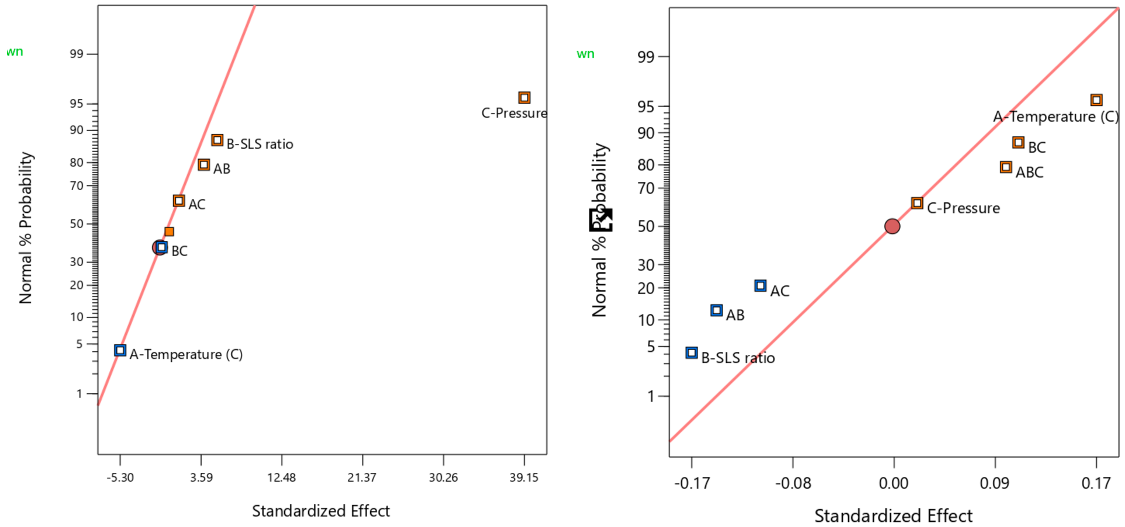

(C) **(d)**

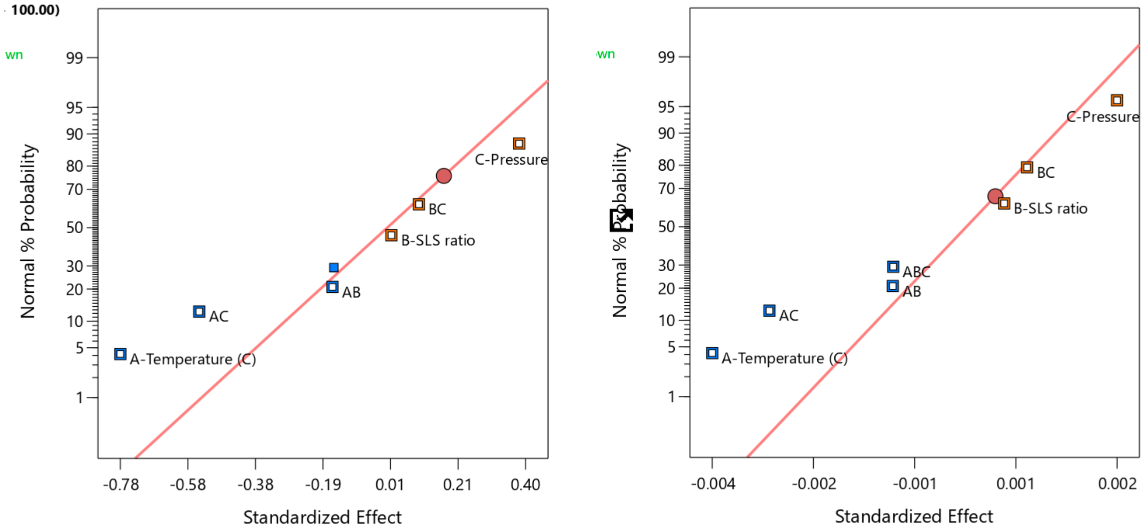

Figure S2: Normal probability plots for (a) Water flux (d) water permeance (c) creatinine residual concentration (d) creatinine removal efficiency developed models

TableS1:. Manufacturers specifications of the pristine dialysate center RO membranes(FilmTec™ HSRO-4040-FF Element.)

Items	RO Membrane
Manufacturer	FilmTec™
Type	HSRO-4040-FF
Membrane material	Thin-Film Composite Polyamide (TFC)
Maximum Operating Temperature1 Maximum	113°F (45°C)
Maximum Sanitization Temperature (@ 25 psi)	185°F (85°C)
Salt rejection (%)	99.0% typical (97.0% minimum)
Max. operating pressure	600 psi (41 bar)
Max. operating temperature	45°C (113°F); above pH 10 limited to 35°C
Operation pH range	2–11
Short-term cleaning pH range	1–12
Active membrane area / Size	~3.3 m^2 (36 ft^2); 4″ diameter × 40″ length

Table S2: RSM models ANOVA for REoLM water flux and permeance

Term	Coefficient	Effects	% Contribution	SE of coefficient	F-Value	p-value
Water Flux, L/m2/hr (R2 =0.9603)						
Model	37.32				36.31	< 0.0001
A-Temperature (C)	-2.65	-5.30	1.61	1.15	3.80	0.0830
B-SLS ratio	2.70	5.39	1.67	1.36	3.94	0.0785
C-Pressure	19.57	39.15	87.98	1.36	207.77	< 0.0001
AB	2.19	3.91	0.878	1.36	2.07	0.1837
AC	0.6501	1.16	0.078	1.52	0.1833	0.6786
BC	-0.4137	-0.74	0.031	1.52	0.0742	0.7914
ABC		0.11	0.00064	1.52		
Curvature	-9.3669		5.04		11.89	0.0073
Lack of Fit			2.33		2.58	0.3075
Water Permeance, L/m2/hr/bar						
Model	0.6832				3.63	0.0456
A-Temperature (C)	0.0847	0.169307	16.17	0.0278	6.62	0.0329
B-SLS ratio	-0.0833	-0.166524	15.64	0.0329	6.41	0.0352
C-Pressure	0.0103	0.020628	0.240	0.0329	0.0983	0.7619
AB	-0.0815	-0.145792	11.99	0.0329	4.91	0.0575
AC	-0.0612	-0.109466	6.760	0.0368	2.77	0.1347
BC	0.0584	0.104553	6.17	0.0368	2.53	0.1507
ABC	0.0527	0.0942284	5.009	0.0368	2.05	0.1899
Curvature		0.218486	26.93	0.0368	11.03	0.0105
Lack of Fit			6.67		1.14	0.5357

Table S3: RSM models ANOVA for REoLM creatinine residual concentration and removal efficiency

Term	Coefficient	Effects	% Contribution	SE of coefficient	F-Value	p-value
			Creatinine Residual, mg/L			
Model	44.86			0.0202	5.51	0.0108
A-Temperature (C)	-0.0934	-0.187	35.210	0.0239	15.27	0.0029
B-SLS ratio	0.0025	0.00502	0.025	0.0239	0.0110	0.9184
C-Pressure	0.0470	0.0939	8.91	0.0239	3.87	0.0777
AB	-0.0220	-0.0394	1.57	0.0267	0.6808	0.4285
AC	-0.0743	-0.1329	17.84	0.0267	7.74	0.0194
BC		0.0239	0.577			
ABC		-0.039	1.502			
Curvature		-0.137	18.99		8.21	0.0168
Lack of Fit			15.42		5.51	0.0108
			Creatinine Removal, %			
Model	0.0124			0.0004	5.51	0.0142
A-Temperature (C)	-0.0018	-0.00369	33.92	0.0004	17.56	0.0030
B-SLS ratio	0.0003	0.00054	0.742	0.0004	0.3840	
C-Pressure	0.0011	0.002184	11.88	0.0004	6.15	0.0382
AB	-0.0006	-0.001071	2.85	0.0005	1.48	0.2588
AC	-0.0016	-0.00286	20.37	0.0005	10.54	0.0117
BC	0.0005	0.00087	1.982	0.0005	0.9946	0.3478
ABC	-0.0006	-0.001063	2.82	0.0005	1.46	0.2618
Curvature		-0.002273	12.86		6.66	0.0326
Lack of Fit			12.626			

Table S4. Rehabilitated EoL membrane performance numeral optimization goals, results, and desirability for the different target scenarios

**Operational Variables**	**Scenario Variable Target Goals**						**Scenario Optimization Results**					
Name	Scenario 1	Scenario 2	Scenario 3	Scenario 5	Scenario 6	Scenario 7	Scenario 1	Scenario 2	Scenario 3	Scenario 5	Scenario 6	Scenario 7
A: Temperature (C)	is in range	is in range	Maximize	Minimize	Maximize	Maximize	65	65	65	25	45	65
B: SLS ratio	is in range	is in range	is in range	Minimize	45 °C	Maximize	75	75	75	25	40.712	75
C: Pressure	is in range	minimize	minimize	minimize	minimize	Maximize	550	353.69	353.4	276.32	295.71	550
Y_1 Water Flux	maximize	maximize	maximize	maximize	maximize	maximize	59.363	39.92	39.89	32.334	34.82	59.363
Y_2 Water Permeance	maximize	maximize	maximize	maximize	maximize	maximize	1.545	1.7	1.704	1.792	1.775	1.545
Y_3 Creatinine Residual Conc.	minimize	minimize	minimize	minimize	minimize	minimize	0	2.27	2.273	14.596	11.512	0
Y_4 Removal	maximize	maximize	maximize	maximize	maximize	maximize	100	100	97.3	80.165	83.74	100
**Desirability**	-	-	-	-	-	-	**0.718**	**0.586**	**0.64**	**0.604**	**0.585**	**0.827**
